# Serum Lipid Profiles and Cancer Risk in the Context of Obesity: Four Meta-Analyses

**DOI:** 10.1155/2013/823849

**Published:** 2013-01-20

**Authors:** Jennifer C. Melvin, Lars Holmberg, Sabine Rohrmann, Massimo Loda, Mieke Van Hemelrijck

**Affiliations:** ^1^Cancer Epidemiology Group, Division of Cancer Studies, King's College London, School of Medicine, 3rd Floor, Bermondsey Wing, Guy's Hospital, London SE1 9RT, UK; ^2^Regional Cancer Centre, Uppsala University Hospital, 751 85 Uppsala, Sweden; ^3^Department of Surgical Sciences, Uppsala University, 751 85 Uppsala, Sweden; ^4^Department of Epidemiology and Prevention of Cancer, Institute of Social and Preventative Medicine, University of Zurich, 8001 Zurich, Switzerland; ^5^Department of Medical Oncology, Dana-Farber Cancer Institute, Boston, MA 02215, USA; ^6^Department of Pathology, Brigham and Women's Hospital of Harvard Medical School, Boston, MA 02115, USA; ^7^Broad Institute of Harvard and MIT, 7 Cambridge Center, Cambridge, MA 02142, USA; ^8^Division of Cancer Studies, School of Medicine, King's College London, 3rd Floor, Bermondsey Wing, Guy's Hospital, London SE1 9RT, UK

## Abstract

The objective here was to summarize the evidence for, and quantify the link between, serum markers of lipid metabolism and risk of obesity-related cancers. PubMed and Embase were searched using predefined inclusion criteria to conduct meta-analyses on the association between serum levels of TG, TC, HDL, ApoA-I, and risk of 11 obesity-related cancers. Pooled relative risks (RRs) and 95% confidence intervals were estimated using random-effects analyses. 28 studies were included. Associations between abnormal lipid components and risk of obesity-related cancers when using clinical cutpoints (TC ≥ 6.50; TG ≥ 1.71; HDL ≤ 1.03; ApoA-I ≤ 1.05 mmol/L) were apparent in all models. RRs were 1.18 (95% CI: 1.08–1.29) for TC, 1.20 (1.07–1.35) for TG, 1.15 (1.01–1.32) for HDL, and 1.42 (1.17–1.74) for ApoA-I. High levels of TC and TG, as well as low levels of HDL and ApoA-I, were consistently associated with increased risk of obesity-related cancers. The modest RRs suggest serum lipids to be associated with the risk of cancer, but indicate it is likely that other markers of the metabolism and/or lifestyle factors may also be involved. Future intervention studies involving lifestyle modification would provide insight into the potential biological role of lipid metabolism in tumorigenesis.

## 1. Introduction

Obesity is a major worldwide problem, over 30% of adults in Western populations are obese, and there is growing evidence of the associated health risks associated [1–5]. The link between obesity and cancer risk has been studied extensively, but the results of individual studies do not suggest a consistent association [1, 6, 7]. Common cancers studied in the context of obesity include colorectal, breast, prostate, endometrial, pancreatic, liver, ovarian, kidney, gallbladder, leukaemia, and oesophageal cancers [4, 7–17].

The underlying mechanisms of action are not clear [1, 18–20]. A solid understanding could translate directly to patient benefit through implementation of therapeutic strategies to reduce cancer risk and mortality [19]. Assuming that the lipid metabolism plays a role in the biological processes driving the development of cancer, this could be easily modified by existing methods such as exercise, medication, or diet. Increased physical activity levels improve cardiovascular and overall mortality in healthy populations [21–24]. Also, physical activity after cancer diagnosis is associated with improvements in cancer outcomes [25, 26] and metabolic markers such as cholesterol [27, 28]. As such, improvements in lipid levels through uptake of physical activity may also translate into improvements in cancer-specific survival. Experimental evidence largely suggests that statins, a commonly used drug to lower cholesterol levels, reduce cancer risk, though further trials are needed [29]. However, a large meta-analysis by the Cholesterol Treatment Trialists' Collaboration showed no statistically significant associations between statin use and cancer risk [30]. Nevertheless, due to heterogeneity of plasma lipid profiles in overweight and obese people, there may be some inconsistency in the associations for serum lipids and cancer risk [31]. Moreover, study populations were often small, insufficient information was collected (e.g., lack of BMI measurement, few lipid components measured), and timing of blood sampling in relation to diagnosis varied widely [32, 33]. In addition, it is thought that tissue types are influenced differently by lipid components and to varying degrees [2, 32]. 

With these meta-analyses, we aimed to summarize and quantify the evidence for the link between markers of lipid metabolism and risk of obesity-related cancers. We examined the associations between four components of the lipid profile measured in serum (total cholesterol (TC), triglycerides (TGs), high-density lipoprotein (HDL), and apolipoprotein A-I (ApoA)) and risk of cancers previously shown to be linked with obesity.

## 2. Methods

### 2.1. Literature Search Strategy

We used computerised literature search databases (PubMed search followed by an Embase search) to identify full text and abstracts published to date. Searches were conducted both with and without MeSH terms (“neoplasms/epidemiology,” “cancer,” “hyperlipidemias,” “lipoproteins, HDL,” “hypertriglyceridemia,” “lipoproteins, apo A”). Except for English language, human subjects, adults, and publications within the last 10 years no additional restrictions were added to the search. We also included “grey literature,” such as letters and abstracts presented in relevant conference meetings. All references of the selected articles were checked, including hand searches.

### 2.2. Inclusion Criteria

**Figure 1:**
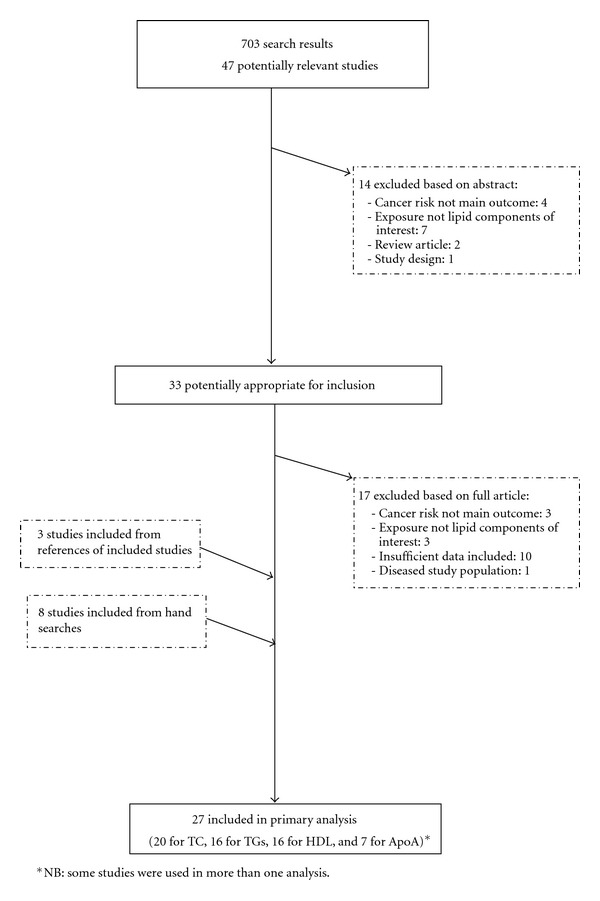


The final collection of selected studies was chosen based on the following criteria: the publication pertained to an epidemiological study (cohort or case-control studies), which measured the serum concentration of at least one of the selected lipid components (TC, TGs, HDL, and ApoA-I) prior to cancer diagnosis; the analytical methods were well described, with sufficient data available; the cancers included must have previously been linked to an increased risk associated with obesity. Those included were colorectal, breast, prostate, endometrial, pancreatic, liver, ovarian, kidney, gallbladder, leukaemia, and oesophageal cancer [7]. To include studies with large enough power, only those with at least 20 cancer cases were included. Initially, titles and abstracts of articles were reviewed in order to ascertain whether they potentially fit the inclusion criteria. If there was doubt over whether an article met the relevant criteria, it was subjected to a thorough assessment. After this first selection, all articles underwent detailed evaluations of the methods and results.  Figure 1 illustrates the study exclusion process.

### 2.3. Data Extraction

The following details were recorded for each study: author, year of publication, country where the study was undertaken, serum lipid component levels (mmol/l), study type (case-control or cohort), cancer type, number of cases and total subjects for each level of the lipid component(s) measured. To allow for ease of comparison, all values in conventional units (mg/dL) were converted into SI units (mmol/l) using conversion factors [34].

### 2.4. Statistical Methods

The effect of each lipid component on cancer risk was evaluated by calculating the random effects summary relative risk to allow for possible heterogeneity between study results. The analyses were conducted for dichotomized values of TC, TGs, HDL, and apoA-I). The following clinical cutpoints were used TC ≥ 6.50; TG ≥ 1.71; HDL ≤ 1.03; ApoA-I ≤ 1.05 mmol/L, all of which mirrored the NCEP and WHO guidelines as closely as practicable [35–39]. Some studies presented with dichotomised serum lipid levels, but for those that did not, participants from each study were divided into two groups based on their serum lipid level (“high” and “low”), and this mirrored the NCEP and WHO guidelines as closely as practicable. A first meta-analysis used all cancers from all studies. Potential heterogeneity of the study results was assessed with weighted forest plots, which display the relative risk estimates of cancer risk for each lipid component. Heterogeneity of the study results was also statistically evaluated using the *Q*-statistic as well as the *I* ^2^ statistic [40]. Sufficient data allowed for an individual analysis (using the methods described above) of prostate, colorectal, and breast cancers. Finally, we performed a metaregression to evaluate the effect of study design (i.e prospective cohort versus case-control studies). No other potential confounders were included in the metaregression due to the nature in which the data was presented through the papers. For example, some papers acknowledge having data for fasting status of the individuals at time of sampling, but did not provide the actual data by individuals. Potential publication bias and effect modification (by country and year) were assessed using Begg's Test and Egger's funnel plot. All analyses were performed using STATA (version 11.2).

## 3. Results

The initial searches produced a total of 701 articles. 33 studies were selected for further evaluation based on information from abstracts. Of these, 17 were excluded and a further 12 were added from hand searches and references of included studies, giving a total of 28 studies used in the primary data analysis. Eight studies were conducted in Asia, 12 in Europe, and eight in the USA (Table 1). All studies reported on at least one of the four lipid components under investigation. The nine included cancers were studied in association with cancer risk; all cancer diagnoses were histologically confirmed. Of those articles examined for inclusion, the major reasons for exclusion were missing information on methods and statistical analysis (*n* = 10), while a further three were excluded because serum lipid components were not the exposure of interest, and three were removed because incident cancer risk was not the outcome variable in the analysis.

**Figure 2:**
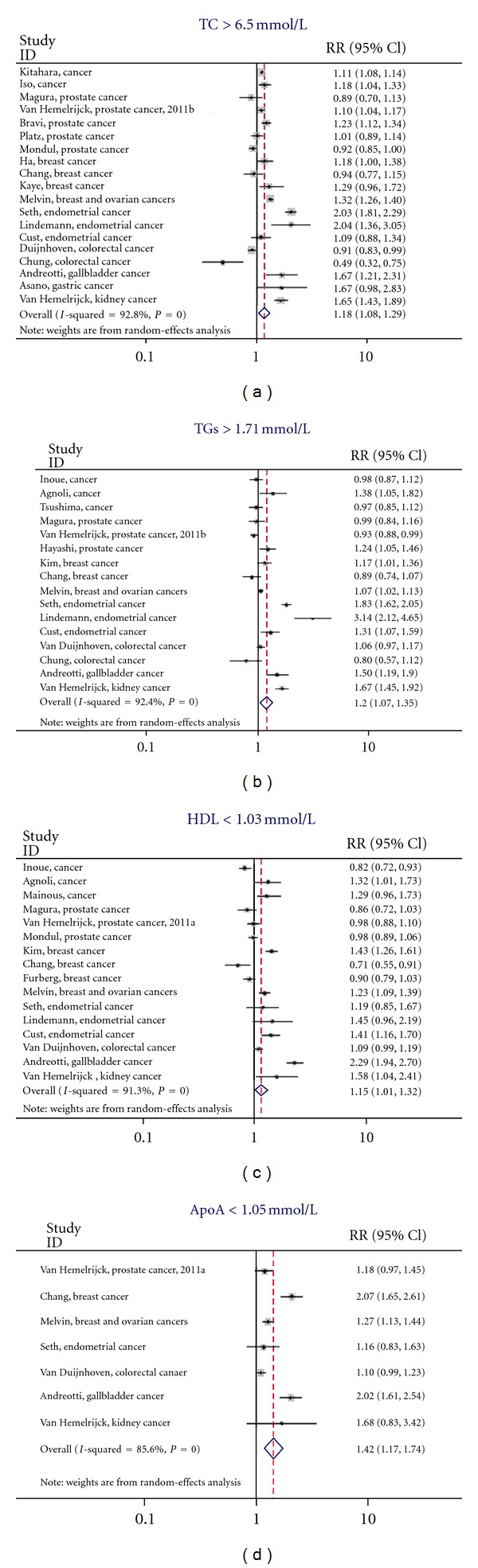
Individual forest for lipid components, the *I*-squared statistic is also illustrated in each plot—total cholesterol; triglycerides; high-desity Lipoprotein; apolipoprotein A-I.

For each lipid component studied we looked at the risk of developing cancer in those with abnormal versus normal levels. The random-effects analysis, comparing overall cancer risk and total cholesterol level, showed a pooled effects relative risk of 1.18 (95% CI 1.08–1.29) (Figure 2). The *Q*-statistic and *I* ^2^-statistic suggested heterogeneity (*Q* = 250.02; d*f* = 18; *P* = <0.000; *I* ^2^ = 92.8%), which warranted the use of a random-effects model. The association between TGs and overall cancer risk resulted in a pooled relative risk of 1.20 (95% CI 1.07–1.35). The pooled relative risk was 1.15 (95% CI 1.01–1.32) when studying the association between HDL and risk of obesity-related cancers. The pooled effects relative risk of overall cancer was 1.42 (95% CI 1.17–1.74) for ApoA-I (Figure 2).

**Figure 3:**
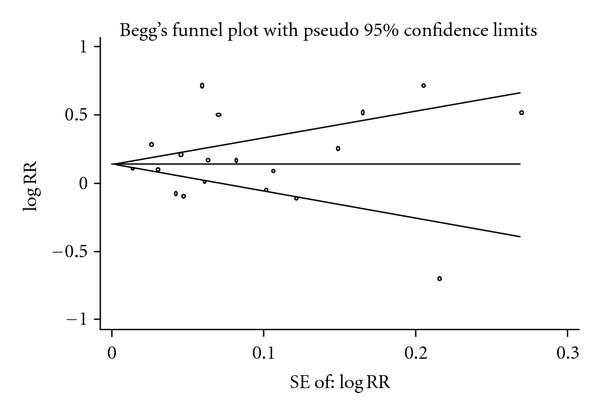
Funnel plot of Begg's test (with 95% CI) to quantify presence of publication bias.

We also conducted a stratified analysis by study type and found that the pooled RRs for case-control studies were slightly different (i.e., RR (95% CI) = 0.98 (0.81–1.18)) and (RR (95% CI) = 1.20 (1.04–1.38) for case-control and cohort studies on TC, resp.). We investigated this further with a metaregression, but did not find a statistically significant effect (i.e., *P* value of 0.193 when studying TC). Begg's test did not indicate significant publication bias (*P* = 0.506), which is evident from the funnel plot, as there is a relatively symmetric distribution observed among studies with small sample size (Figure 3).

Finally, we also performed a meta-analysis specifically for TC and risk of prostate, breast, and colorectal cancer. The pooled relative risk for prostate cancer was 1.04 (95% CI: 0.87–1.24), whereas it was 1.08 (95% CI: 0.89–1.31) and 1.20 (95% CI: 0.44–3.26) for breast and colorectal cancers, respectively.

## 4. Discussion

These meta-analyses summarize the current evidence for a link between serum markers of lipid metabolism and risk of obesity-related cancers. All pooled models showed evidence for an association between abnormal lipid components and risk of obesity-related cancers when using clinical cutpoints. 

The precise aetiology of the link between obesity and risk of cancer has yet to be determined, but there has been growing evidence for a role of lipid metabolism in tumour development [18]. Apart from the studies listed on the link between serum lipids and cancer risk (Table 1), there is also preclinical evidence. For instance, it is thought that androgens stimulate prostate tumor growth via activation of pathways that regulate lipogenic gene expression, resulting in lipid accumulation [41]. Hyperlipidemia has also been shown to be involved in colorectal tumour development and initiation and progression of breast and prostate cancers [42–44]. Moreover, there is experimental evidence that fatty acid synthase (FAS), the enzyme that synthesizes fatty acids *de novo*, is involved in tumorigenesis [45–47]. For example, prostate cancers overexpressing FAS display aggressive behavior, with the highest expression in patients with bony metastatic disease [47, 48]. In addition, nutritional studies showed that diets high in fat are linked to accelerated tumour growth and metastasis [42, 49]. Furthermore, cholesterol-lowering drugs such as statins have been shown to reduce the formation and spread of metastatic cancer cells [50, 51]. Finally, the immune system is thought to play a role in the link between HDL, ApoA-I, and tumorigenesis [52]. These lipid components decrease free proinflammatory cytokines such as tumour necrosis factor-*α* (TNF-*α*) which consequently reduces tissue damage, infiltration of macrophages and neutrophils, and attenuates tumour formation [53]. Therefore, low levels of HDL and Apo A-I may contribute to an inflammatory process linked to tumour biology. 

Recent years have seen a multitude of reviews and meta-analyses comparing obesity and specific cancer risks; results varied widely depending on the type of cancer investigated, with relative risks ranging from 1.02 to 4.10 (breast cancer [11, 54–56], endometrial cancer [57], pancreatic [58–60], liver [17], prostate cancer [61], and colon and rectal cancer [15, 16]). As a result, our findings for an association between serum lipid components and risk of cancer also varied by type of cancer, as can be seen from our results for HDL (Figure 2), show that there may be stronger correlation between serum HDL levels and cancer risk, dependent on cancer type. Those results focusing on a single cancer showed more consistent results, suggesting that even among obesity-related cancers there may be a different association with serum lipid levels.

### 4.1. Strengths and Limitations of This Study

The greatest strength of this study is that we examined four different components of the lipid profile in relation to risk of developing cancer in the context of obesity. We also made all possible efforts to include all relevant available publications, including searching the two main online databases (PubMed and Embase). Additionally, our clearly defined objective criteria for exposure, outcome, and other study characteristics were specified a priori. There was no evidence of publication bias in these analyses.

A number of the studies subdivided levels of lipid components, but this was not performed consistently across the studies. Studies which had not dichotomised serum lipid levels from the outset were divided into two groups based on their serum lipid level (“high” and “low”) to mirror the NCEP and WHO guidelines as closely as practicable [38, 62]. This crude categorization may have compromised the accuracy and resulted in miscategorising of individuals, but given the rather small differences in cutoffs we do not believe that this has had a major impact on our analyses.

Heterogeneity among studies may also arise from different method of assessment of serum lipids. By performing random-effects analyses, we have taken into account between-study variation. Within-person variation is a likely interference with results as the one measurement taken may not be representative for a person's average, or previous lipid levels. However, this variation will be present in all studies using a single measurement. In addition, adjustments made for confounding factors (e.g., gender or age) were not consistent across included studies and some sample sizes were relatively small or excluded one gender. Again, random effects analyses take into account this heterogeneity and in addition we included a metaregression analysis for study type. 

In addition, the studies did not provide age-specific data, so it was not possible to conduct age-specific meta-analyses which presents us with a limitation. Persons younger than middle aged more rarely have abnormal lipid profiles and are also considerably less likely to be diagnosed with the cancers of interest than in those people aged over 50. Thus, this leaves our study population with a relatively low probability of having both sufficient exposure and number of cases in the lower age range. We do not believe that this will have had a major effect on our results, although it is worth considering that this may have diluted the strength of our findings somewhat. 

Due to the information provided in the included studies, we had no means to adjust our analyses for cancer screening practices. Undoubtedly, these practices vary around the world and thus the differences could lead to the introduction of detection bias. 

Finally, the analyses of the three individual cancers (prostate, breasts and colorectal cancers) did not produce statistically significant relative risks, which most likely follows from a lack of power due to the limited number of studies available for inclusion. Future research, with larger sample sizes, repeated measurements, and consistent adjustments for confounding could provide information to inform a more reliable estimate of links between serum lipid components and cancer risk. 

### 4.2. Conclusions

Abnormal levels of all lipid components studied were statistically significantly associated with an increased risk of obesity-related cancers, with the strongest association for serum ApoA-I. Despite a suggestion for a link between the lipid metabolism and risk of cancer, the magnitude of the pooled relative risk was relatively small. This may be because the studied lipid components are markers of obesity or because they are markers of other lifestyle factors potentially associated with tumorigenesis. Since lipid components are easily modified through lifestyle interventions such as diet or exercise, research into serum lipid components and cancer risk presents a prime opportunity for intervention studies to help provide the desired insight into their biological role.

## Figures and Tables

**Table 1:** Details of included studies.

Author (y)	Country	Cases/controls	Study Type	Cancer(s) included	Mean age (SE)/age range	Timing of measurement	Measurement method	Cholesterol measure	Adjusted for
Inoue et al. (2009) [63]	Japan	1263/26461	Cohort	Colon/rectum and breast	56.0 (8.2)	Fasting and nonfasting	NS	TGs, HDL	Adjusted for age, study area, smoking status, weekly ethanol intake, and total serum cholesterol
Iso et al. (2009) [64]	Japan	1093/32275	Cohort	Colorectal, prostate, leukemia, breast and cervical	54.0	Fasting and nonfasting	Enzymatic	TC	Age, BMI, pack year of smoking, ethanol intake, hypertension, diabetes, hyperlipidemia medication use, total vegetable intake, coffee intake, and public health centre
Kitahara et al. (2011) [65]	Korea	44935/ 1144784	Cohort	Oesophagus, colon/rectum, pancreas, prostate, kidney, and gallbladder	30–95	Fasting	NS	TC	Cigarette smoking, alcohol consumption, BMI, physical activity, hypertension, and fasting serum glucose
Mainous et al. (2005) [66]	USA	203/3075	Cohort	Cancer	30+	12 hr fasting	NS	HDL	Age, gender, smoking status, and BMI
Melvin et al. (2012) [67]	Sweden	6871/ 227603	Cohort	Breast and ovarian	25+	Fasting and nonfasting	Enzymatic and immunoturbidimetric	TC, TGs, HDL, ApoA	Glucose, TGs, TC, age, parity fasting status, and SES
Chang et al. (2007) [68]	Turkey	150/71	Case	Breast	49.2 (11.8)	12 hr fasting	Enzymatic	TC, TGs, HDL, ApoA	HDL, apoA-I, apoB, ApoA-I/ApoB ratio, and VLDL
Furberg et al. (2004) [69]	Norway	1287/ 27912	Cohort	Breast	43.6 (0.1)	Nonfasting	Enzymatic	HDL	Age, country of residence, parity, height, TC, recreational and occupation activity. Some models also included blood pressure, BMI, TGs, age at first birth, time since last meal, dietary energy, and fat intake
Ha et al. (2009) [70]	Korea	714/169660	Cohort	Breast	55.9 (5.0)	Fasting	NS	TC	Age and BMI
Hayashi et al. (2012) [71]	Japan	377/528	Case		>60	Fasting	NS	TGs	Age, PSA level, prostatic volume, BMI, and TGs level
Kaye et al. (2002) [72]	USA	158/725	Case	Breast	50–79	NS	NS	TC	NS
Kim et al. (2009) [73]	Korea	690/1380	Case	Breast	48.5 (7.6)	8 hr fasting	Enzymatic	TGs, HDL	HDL, age, family history of breast cancer, age at menarche, age at the first full-term pregnancy, and total cholesterol
Lindemann et al. (2009) [74]	Norway	100/31273	Cohort	Endometrial	56.1	Nonfasting	Enzymatic	TC, TGs, HDL	Age, other lipids, and BMI
Cust et al. (2007) [75]	France	284/546	Case	Endometrial	56.9	Fasting and nonfasting	Enzymatic	TC, TGs, HDL	Age, laboratory batch, and case-control status
Seth et al. (2012) [76]	Sweden	1144/ 224288	Cohort	Endometrial	25+	Fasting and nonfasting	Enzymatic and immunoturbidimetric	TC, TGs, HDL, ApoA	Glucose, TC, TGs, age, parity, fasting status, and SES
Magura et al. (2008) [77]	USA	312/319	Case	Prostate	50–74	NS	NS	TC, TGs, HDL	Age, family history of prostate cancer, BMI, type 2 diabetes, smoking, and multivitamin use
Mondul et al. (2010) [78]	USA	438/6378	Cohort	Prostate	35+	NS	Enzymatic	TC	Age, race, BMI, education level, smoking status, intake of meat, dairy, tomato products and alcohol, family history of prostate cancer, PSA screening, and use of diabetes medications
Mondul et al. (2011) [79]	USA	2041/27052	Cohort	Prostate	50–69	NS	Enzymatic	TC, HDL	Age, serum *α*-tocopherol, family history of PCA, education level, and urban residence
Platz et al. (2009) [80]	USA	1251/4335	Cohort	Prostate	63.1	Nonfasting	Enzymatic	TC	Age, race, first-degree family history of prostate cancer, BMI, self-reported diabetes mellitus, regular aspirin use, and history of myocardial infarction
Van Hemelrijck et al. (2011) [81]	Sweden	2008/65719	Cohort	Prostate	35+	Fasting and nonfasting	Enzymatic and immunoturbidimetric	HDL, ApoA	Age, glucose, TGs, and TC, fasting status, and SES.
Van Hemelrijck et al. (2011) [82]	Sweden	5112/ 195548	Cohort	Prostate	45–75	Fasting and nonfasting	Enzymatic and immunoturbidimetric	TC, TGs	Glucose and/or TGs and/or TC, SES, fasting status, and time between measurements and entry
Bravi et al. (2005) [83]	Italy	1294/1451	Case	Prostate	46–74	NS	NS	TC	Age, centre, education, BMI, physical activity, tobacco smoking, alcohol consumption, and family history of prostate cancer
Tsushima et al. (2005) [84]	USA	1004/21255	Cohort	Colorectal	NS	Nonfasting	NS	TGs	Age, elapsed time since last calorific intake and 50 g glucose load, BMI, heart rate, cigarette smoking history, alcohol intake, and 24 h intake of total calories
Van Duijnhoven et al. (2011) [85]	W. Europe	939/939	Cohort	Colorectal	35–70	Fasting and nonfasting	Enzymatic	TC, TGs, HDL, ApoA	Age, gender, BMI, other lipids, height, weight, smoking habits, physical activity, education, consumption of fruit, vegetables, meat, fish, and alcohol, intake of fibre, energy from fat, and energy from nonfat
Chung et al. (2006) [86]	Korea	105/105	Case	Colorectal	58.6 (8.3)	12 hr fasting	Enzymatic	TC, TGs	Age, gender, BMI, glucose, triglycerides, and total cholesterol
Van Hemelrijck et al. (2012) [87]	Sweden	156/82391	Cohort	Kidney	20+	Fasting and nonfasting	Enzymatic and immunoturbidimetric	TC, TGs, HDL, ApoA	Age, gender, glucose, TGs and TC, creatinine levels, fasting status, and SES
Andreotti et al. (2008) [88]	USA	264/1839	Case	Biliary	34–75	Overnight fasting	NS	TC, TGs, HDL, ApoA	Age, gender, BMI, waist-to-hip ratio, cigarette smoking, alcohol drinking, hypertension, diabetes, and gallstone status
Asano et al. (2008) [89]	Japan	97/2507	Cohort	Gastric	59.2 (0.3)	Fasting and nonfasting	Enzymatic	TC	Age and gender

NS: not specified; BMI: body mass index; TC: total cholesterol; TGs: triglycerides; HDL: high-density lipoprotein; ApoA: apolipoprotein A-I; ApoB: apolipoprotein B; VLDL: very low-density lipoprotein.

## Abbreviations

(TC): Total cholesterol
(TGs): Triglycerides
(HDL): High-density lipoprotein
(ApoA-I): Apolipoprotein A-I
(BMI): Body mass index.
